# Cholinesterase activity in serum during general anesthesia in patients with or without vascular disease

**DOI:** 10.1038/s41598-021-96251-5

**Published:** 2021-08-17

**Authors:** Yitzhak Brzezinski-Sinai, Ester Zwang, Elena Plotnikova, Ester Halizov, Itzhak Shapira, David Zeltser, Ori Rogowski, Shlomo Berliner, Idit Matot, Shani Shenhar-Tsarfaty

**Affiliations:** 1 Division of Anesthesia, Intensive Care and Pain Medicine, Tel Aviv Medical Center, Weizmann 6 St, Tel-Aviv, Israel; 2 Departments of Internal Medicine “C”, “D” and “E”, Tel-Aviv Sourasky Medical Center, Sackler Faculty of Medicine, Tel-Aviv University, Weizmann 6 St, Tel Aviv, Israel

**Keywords:** Predictive markers, Cardiovascular biology

## Abstract

Maintaining hemodynamic stability during the induction and maintenance of anesthesia is one of the challenges of the anesthesiologist. Patients with vascular disease are at increased risk of instability due to imbalance between the sympathetic and parasympathetic parts of the autonomic nervous system, a balance accessible by serum cholinesterase activity. We aim to characterize the dynamics of cholinesterase activity in patients undergoing general anesthesia (GA) and surgery. This was a prospective study of 57 patients undergoing ambulatory or vascular surgery under GA. Cholinesterase activity was measured before the induction of anesthesia, after 15 min and at the end of surgery by calculating the capacity of serum acetylcholinesterase (AChE) and butyrylcholinesterase to hydrolyze AcetylThioCholine. Data on atherosclerotic disease, anesthesia management were analyzed. Both AChE and total cholinergic status (CS) decreased significantly after GA induction at 15 min and even more so by the end of surgery. Vascular surgery patients had lower baseline cholinesterase activity compared to ambulatory surgery patients. Patients requiring intraoperative administration of phenylephrine for hemodynamic support (21.1%) had a significantly lower level of AChE and CS compared to untreated patients. Our findings serve as a mirror to the sympathetic/parasympathetic imbalance during GA, with a marked decrease in the parasympathetic tone. The data of a subgroup analysis show a correlation between low cholinesterase activity and an increase in the need for hemodynamic support.

## Introduction

Hemodynamic shifts are common during anesthesia and are in part a result of ANS changes due to anesthetic drugs, which blunt sympathetic pathways and increase parasympathetic dominance in a dose-dependent manner. Controlling these hemodynamic changes is a critical task of the anesthesiologist^1,2^. During GA, the function of the autonomic nervous system (ANS) is assessed by indirect measurements such as blood pressure, heart rate, body temperature, and plethysmography^3^.

Patients who suffer from severe atherosclerosis and, specifically, from coronary atherosclerosis, may further challenge the anesthesiologist. These patients present elevated blood pressure, increased resting heart rate and heart rate variability^1,4^, and they are considered to be high-risk candidates for surgery compared to patients without atherosclerosis. However, very little is known about their ANS imbalance, and if it has an effect upon intraoperative or postoperative care or upon morbidity and mortality.

Cholinergic status is determined by the equilibrium of production and degradation of acetylcholine (ACh). The process of degradation in the central and peripheral ANS is done by acetylcholinesterase (AChE) and butyrylcholinesterase (BChE)^5^. Although acetylcholine is the natural substrate of AChE, one study suggested that BChE influences ACh degradation more than AChE, perhaps due to its higher quantity in the plasma^6^. The physiologic role of BChE, however, remains unclear, although the common hypothesis is that it has a protective effect against different toxins^7^.

A correlation was recently found between the activity of AChE and BChE to the resting heart rate, systolic and diastolic blood pressure, cholesterol, body mass index (BMI), inflammatory biomarkers, metabolic syndrome characteristics and heart rate recovery in healthy adults^8–10^. Additionally, repeated measurements of BChE activity during 5 years show little variation in healthy adults^11^. In contrast, such as myocardial infarction or stroke, decreased levels of both cholinesterase which were associated with poor prognosis had been observed following acute events^12^.

Furthermore, there are accumulating data on how the immune system mediates an indirect association between the kidney and nervous system via the cholinergic anti-inflammatory pathway (CAP). Acute kidney injury (AKI) attenuation was achieved via CAP activation induced by vagus nerve stimulation or ultrasound treatment in animal models, although the exact mechanism remains unclear^13,14^.

Data on the effects of anesthesia and surgery on cholinesterase activity are limited. During general anesthesia, the anesthesiologist uses various drugs that competitively inhibit the activity of cholinesterase, among them neostigmine, tramadol, metoclopramide and pancuronium^7,15^. In addition, a recent study found that cholinesterase activity could be related to postoperative delirium^16^.

The rationale of the current study was to assess the ability of serum cholinesterase activity as an indirect measurement of the sympathetic/parasympathetic balance during anesthesia. We aimed to assess the effect of general anesthesia and surgery on cholinesterase activity and to evaluate whether patients with overt atherosclerotic disease demonstrate altered activity levels before and during anesthesia compared to healthy adults without known vascular disease. We also sought to examine the association between enzyme activity and hemodynamic stability during general anesthesia.

## Materials and methods

### Study population

This is a comparative prospective study. It was approved by The Tel -Aviv Sourasky Helsinki committee and conformed to the principles outlined in the declaration of Helsinki (19-0342-TLV). Informed consent was obtained from all participants.

Two groups of patients undergoing surgery under general anesthesia of longer than one hour were enrolled. The first group of patients had known atherosclerotic disease (coronary, peripheral vascular or cerebral) and were scheduled for vascular surgery. The second group had no known atherosclerotic disease and were scheduled for ambulatory surgery. All of the study patients were enrolled consecutively from November 1, 2019 to December 30, 2019.

Exclusion criteria: adult patients with a medical history of Parkinson or Alzheimer’s disease, known muscle paralysis diseases with characteristics of muscle nerve synapse injury, or acute kidney injury by The 2012 Kidney Disease: Improving Global Outcomes (KDIGO) criteria^17^. The vascular group was defined as patients with a known atherosclerotic comorbidity, including myocardial infraction, carotid artery stenosis, peripheral vascular disease and cerebrovascular disease.

### Cholinesterase activities

Blood samples were taken from all patients at three time points (prior to and 15 min after the induction of anesthesia, and at the end of surgery prior to the administration of a neuromuscular blockade reversal agent (neostigmine). All blood samples were taken from intravenous access in an upper limb, and each sample contained 3-5CC in a serum test tube.

We used the ACh analog acetylthiocholine (ATCh) as a substrate that is hydrolyzed by both ACh degrading enzymes (acetylcholinesterase and butyrylcholinesterase) and that reflects the total serum capacity for acetylcholine hydrolysis, referred to as “cholinergic status” (CS).

Serum samples were frozen at − 80 °C until acetylcholine hydrolysis analysis. Acetylcholinesterase and total cholinesterase activity were assayed in triplicates (total volume of serum 20 µl) in a microtiter plate using an adaptation of the Ellman assay^18^. Hydrolysis of 1 mM acetylthiocholine (ATCh, Sigma) was followed by spectrofluorometry (Spectrafluor Plus, Tecan) at 405 nm. Prior to read, we incubated the samples for 20 min in the dark with (for acetylcholinesterase activity) or without (for total cholinesterase activity) 50 μM tetra isopropyl pyrophosphoramide (iso-OMPA, Sigma), which is a specific butyrylcholinesterase inhibitor. We calculated enzyme activity using 13,600 M/cm as the e405 for 5-thio-2-nitrobenzoate^18,19^.

### Intraoperative management and measurements

As a pilot novel study for the effect of general anesthesia on cholinesterase activity, we chose not to standardized anesthesia method or maintenance of anesthesia (Volatile anesthesia vs intravenous anesthesia) to receive more data for subgroup analysis and future research leads.

### Drugs

For the induction of anesthesia, most of the anesthesiologists in our center chose to use propofol bolus 2–3 mg/kg/ etomidate 0.2 mg/kg, fentanyl bolus of 2–3 mcg/kg and rocuronium 0.6 mg/kg. For the maintenance of anesthesia, the most common regimen was volatile anesthesia consisting of isoflurane given between minimal alveolar concentrations (MAC) 0.8–1.1. Total intravenous anesthesia was induced by continuous propofol infusion of 4–6 mg/kg/h. Analgesia was provided with a remifentanil continuous drip of 0.05–2mcg/kg/min or a fentanyl bolus of 50–100 mcg. A neuromuscular blockade consisting of rocuronium was given during induction and for maintenance according to need.

We ruled out the use of drugs that interfere with cholinesterase activity (neostigmine, metoclopramide, tramadol and pancuronium) before the blood samples were taken.

### Monitoring

All patients had standard American Society of Anesthesiology monitoring, including ECG, capnography, oxygen and gas analyzer, peripheral saturation with plethysmography, and central body temperature. Intraoperative temperature was measured from central body temperature located in mid- esophagus. In cases of total intravenous anesthesia, we used Bi-Spectral Index to measure the depth of anesthesia, with values between 40–60 taken as being acceptable. We use Neuromuscular Transmission (NMT) intraoperative to measure the depth of paralysis and to avoid residual paralysis while extubating patient, achieving a Train of Four (TOF) of 90% strength between the first and the fourth twitch.

### Definition and treatment for hypotension

Our criteria for hypotension were mean arterial pressure levels below 65 mmHg for 2 consecutive measurements in a noninvasive and/or invasive blood pressure monitoring. A hypotension event was treated according to the patient's heart rate; Patients with tachycardia were given phenylephrine (50–100mcg bolus), and patients with bradycardia were given ephedrine (5–10 mg bolus).

Our department policy on antihypertensive drugs recommends continuing medication intake of prescribed drugs with the exception of angiotensin-converting enzyme inhibitors on the morning of surgery.

The primary outcome was the characterization of the dynamics of cholinesterase activity in patients undergoing general anesthesia and surgery. The secondary outcome was the assessment of the association between cholinesterase activity and hemodynamic instability during general anesthesia as reflected by the intraoperative use of vasopressors.

### Sample size calculation

Sample size calculations were done separately based on the primary and secondary outcomes. The secondary option required a larger number of patients and it is presented herein.

To detect a relative risk of 1.3 among patients presenting with elevated cholinesterase activity and assuming an event rate of 20%-35% hemodynamic instability during surgery^20^ will require 55 participants (Epi Info 7 software).

### Statistical analysis

All continuous variables are displayed as means (± standard deviation) for normally distributed variables or median (interquartile range) for variables with abnormal distribution. Categorical variables are displayed as numbers (%) of subjects within each group. The independent-samples t-test or the Mann–Whitney test was performed to test differences in continuous variables between the 2 groups. The Pearson Chi-Square test was performed for comparison of dichotomous or categorical variables. Either the paired-samples t-test or the sign-rank test was employed to compare continuous variables between 2 time-points. The Pearson or Spearman correlation coefficients were used to assess correlations between continuous variables. The repeated-measures general linear model was used to compare continuous variables between 3 time points. A one-way analysis of variance (ANOVA) with a linear contrast was used to compare the CS values between vascular and non-vascular groups for blood pressure measurements. In order to identify possible confounders, a multivariate regression with the use of pressor drugs (sympathomimetics and Norepinephrine) was used controlling for the age, and vascular vs non vascular surgery as covariates. P < 0.05 was considered statistically significant for all analyses. We used IBM SPSS Statistics 24 statistical package (IBM Corporation, Armonk, New York, USA) for all statistical analysis.

## Results

### Patient demographics

Fifty-seven suitable patients were enrolled, of whom 17 (29.8%) underwent vascular surgery (peripheral arterial bypass surgery or carotid endarterectomy) and 40 (70.2%) underwent ambulatory surgery, mainly lumpectomy or laparoscopic cholecystectomy. Their demographics are presented in Table 1. One-third of the cohort suffered from essential hypertension, and almost all of them were treated with angiotensin receptor blocker/angiotensin-converting enzyme inhibitor. Comorbidities associated with atherosclerotic disease were prevalent only in the vascular surgery group. The patients’ comorbidities and medication use are listed in Table 2.

**Table 1:** Demographics, comorbidities & medications.

Patients’ characteristics		Vascular (n = 17)	Non vascular (n = 40)
Age, years, mean(SD)	52.3 (19.1)	71(9)	45 (17)
Gender, % male, n (%)	23 (40.4)	12 (70.5)	11 (27.5)
Body mass index- BMI , kg/m^2^, mean(SD)	25.5 (5.0)	24.9 (3.4)	25.6 (5.3)
Smoking, n (%)	25 (43.9)	11 (64.7)	14 (35)
**Surgical and post-anesthesia care Unit details**			
Department, n (%)			
Vascular	17 (29.8)		
General surgery	26 (45.6)		
Nose Ear Throat	7 (12.2)		
Urology	1(1.75)		
Plastic	6 (10.5)		
**American Society of Anesthesiologist (ASA) Physical Status Classification System, n (%):**			
1	12 (21.1)	0	12 (30)
2	28 (49.1)	2 (11.7)	26 (65)
3	14 (24.6)	11 (64.7)	3 (7.5)
4	3 (5.3)	3 (17.6)	0
Anesthesia length, min, median (IQR)	150 (60–480)	287 (113–480)	117 (60–360)
Post-anesthesia Care Unit length of stay, min, median (IQR)	195.0 (100.5–327.5)	330 (160–420)	126.5 (63–584)
Parameter	n (% total patients)	n (% vascular)	n (% non-vascular)
Essential hypertension	18 (31.6)	13 (76.4)	4 (10)
Myocardial infraction	8 (14)	8 (47)	0
Anemia	9 (15.7)	7 (41.1)	2(5)
Carotid artery stenosis	4 (7)	4 (23.5)	0
Peripheral vascular disease	10 (17.5)	10 (58.8)	0
Diabetes mellitus	7 (12.3)	6 (35.2)	1 (2.5)
Chronic kidney disease	4 (7)	4 (23.5)	0
Tumor	14 (24.5)	4 (23.5)	10 (25)
Depression	5 (8.8)	2 (11.7)	3 (7.5)
Beta blockers	7 (12.3)	6 (35.2)	1 (2.5)
Calcium channel blocker	8 (14)	8 (47)	0
Angiotensin receptor blocker/ Angiotensin converting enzyme inhibitor	15 (26.3)	12 (70.5)	3 (7.5)
Others	11 (19.5)	10 (58.8)	1 (2.5)

Others: Congestive heart failure, cerebrovascular disease, chronic obstructive pulmonary disease, Hematologic tumor, Obstructive sleep apnea.

**Table 2:** Cholinesterase activity at baseline, 15 min after induction of anesthesia and at the end of anesthesia.

	Baseline	15 min after induction of anesthesia	Paired p-value	End of anesthesia	Paired p-value (compared to baseline)	Paired p-value (compared to 15 min)
AChE, nmol/min/ml	453.7 ± 216.8	403.4 ± 201.1	** < 0.001**	384.8 ± 201.5	** < 0.001**	**0.029**
Cholinergic status, nmol/min/ml	1396.2 ± 614.8	1241.1 ± 568.0	** < 0.001**	1165.8 ± 568.9	** < 0.001**	**0.005**

*AChE* measurement achieved after incubating the samples with tetraisopropyl pyrophosphoramide a specific ButyrylCholinestersase inhibitor. Followed by spectrofluorometry using ACh analog-acetylthiocholine (ATCh).

Cholinergic Status (CS) spectrofluorometry using ACh analog- acetylthiocholine (ATCh), without incubating with tetra isopropyl pyrophosphoramide.

### Cholinergic enzyme activity and status

#### Primary outcome

Both AChE and total cholinergic status (CS) decreased significantly after general anesthesia induction at 15 min and even more so by the end of surgery (Table 3). The decrease in enzymatic activity was significant. The mean change (delta, paired format) from baseline to 15 min post-induction of anesthesia was 70.5 ± 91 p < 0.001 and 234.7 ± 278.2 p < 0.001 for AChE and CS, respectively. This finding remained significant after repeated measurements for linear regression adjusted for age, gender, BMI, vascular vs. non-vascular surgery and American Society of Anesthesiologists (ASA) Physical Status Classification System.

**Table 3:** The effect of vasopressor drugs on Cholinesterase activities.

Drug		Baseline	Paired p-value	15 min after induction of anesthesia	Paired p-value	End of anesthesia	Paired p-value
Phenylephrine (n = 12, 21.1%) Vascular n = 9, 52.9% mean 2846.6mcg Non-vascular n = 3, 7.5% mean 125mcg	AChE^a^	364.5 ± 71.9	**0.008**	340 ± 94.72	0.067	300.6 ± 87.8	**0.013**
	AChE^b^	477.1 ± 233.9		420.64 ± 219.07		407.7 ± 217.8	
	CS^a^	1152.5 ± 223.3	**0.011**	1035.42 ± 184.33	**0.019**	935.8 ± 222.5	**0.011**
	CS^b^	1463.0 ± 662.8		1297.25 ± 623.69		1232.5 ± 612.5	
Ephedrine (n = 19, 33%) Vascular n = 9, 52.9% mean = 15.86 mg Non-vascular n = 10, 25% Mean = 13.5 mg	AChE^a^	431.1 ± 119.4	0.496	375.5 ± 138.4	0.529	332.7 ± 174.6	0.167
	AChE^b^	464.7 ± 250.2		417.7 ± 227.1		411.6 ± 211.3	
	CS^a^	1322.9 ± 354.2	0.427	1182.1 ± 412.1	0.393	1006.8 ± 477.9	0.124
	CS^b^	1435.1 ± 704.7		1271.5 ± 636.6		1252.2 ± 592.6	

*AChE* Acetylcholinesterase, *CS* Cholinergic status, both measured in nmol/min/ml.

^a^Treated.

^b^Not treated.

**Figure 1:**
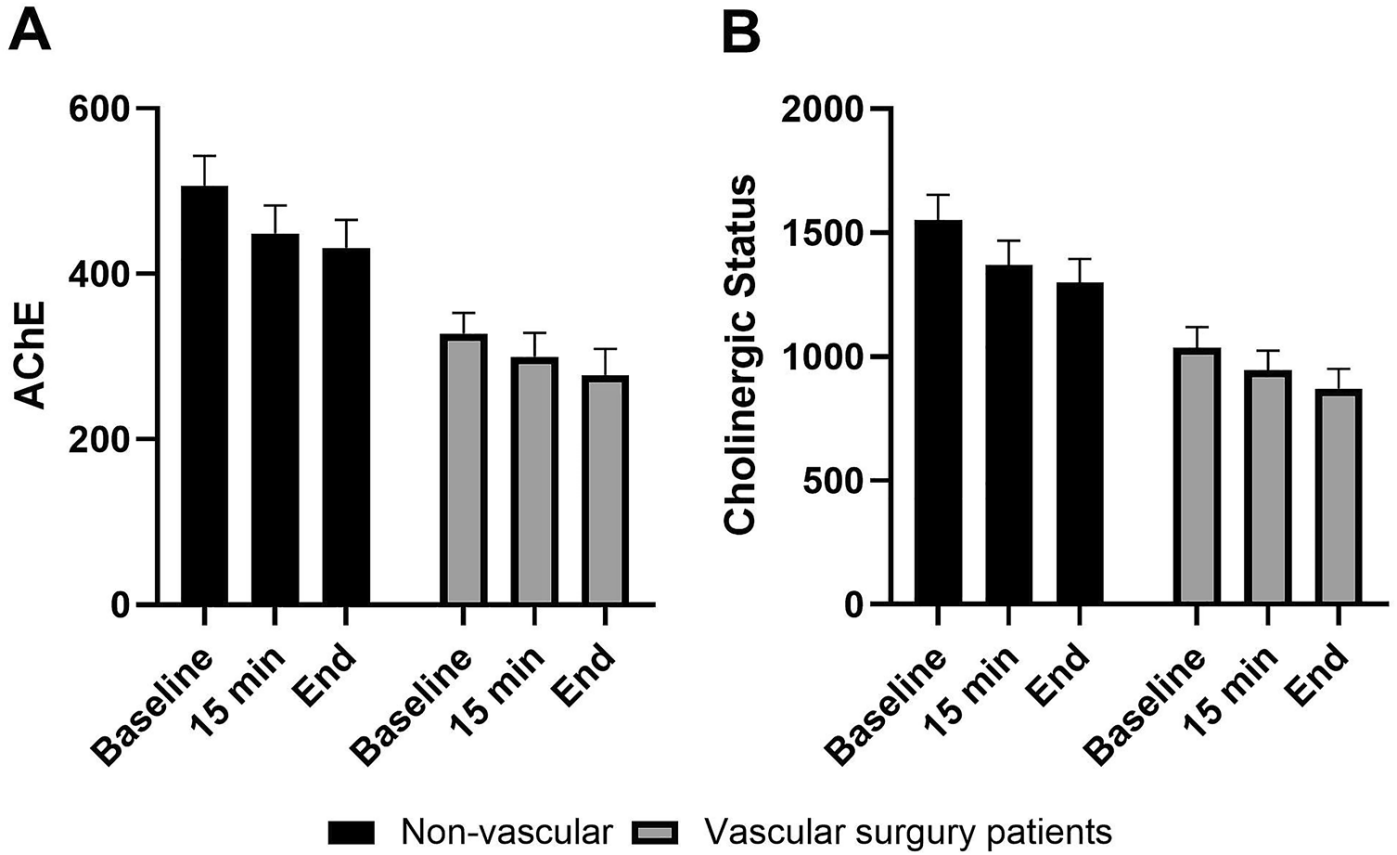
Mean AChE activity (**A**) and total cholinergic status in vascular vs. non-vascular patients by time of measurement during anesthesia (baseline, 15 min and at the end of surgery).

Comparison of the two main groups (vascular surgery patients vs. ambulatory surgery in patients without atherosclerotic disease) revealed that a significant difference of cholinesterase activity had already been present at baseline, with lower activity for the vascular surgery patients (327.9 ± 101.9 vs. 506.8 ± 228.6 for AChE, and 1035.7 ± 343.7 vs. 1551.5 ± 635.1 for CS, respectively p < 0.001 for both). During general anesthesia, significant decreases in both AChE and CS from baseline measurement to 15 min after induction of anesthesia were observed only in the ambulatory surgery group (i.e., patients without known atherosclerotic disease), with further significant reduction by the end of anesthesia compared to baseline measurements (Fig. 1A,B).

#### Secondary outcome

##### Vasopressor support and cholinesterase activities

The association between vasopressor use (reflecting the need to optimize hemodynamics) and cholinesterase activity before and following anesthesia was assessed. Patients who required intraoperative administration of phenylephrine for hemodynamic support (n = 12, 21.1%) had significantly lower activity of AChE and CS at both the beginning and at the end of anesthesia compared to patients not treated with phenylephrine (p = 0.008 and p = 0.013 for AChE and p = 0.011 and p = 0.011, respectively). There were no differences in enzyme activity at any of the time points measured between the patients who received ephedrine (n = 19, 33.3%) and those who did not.

##### Temperature

**Figure 2:**
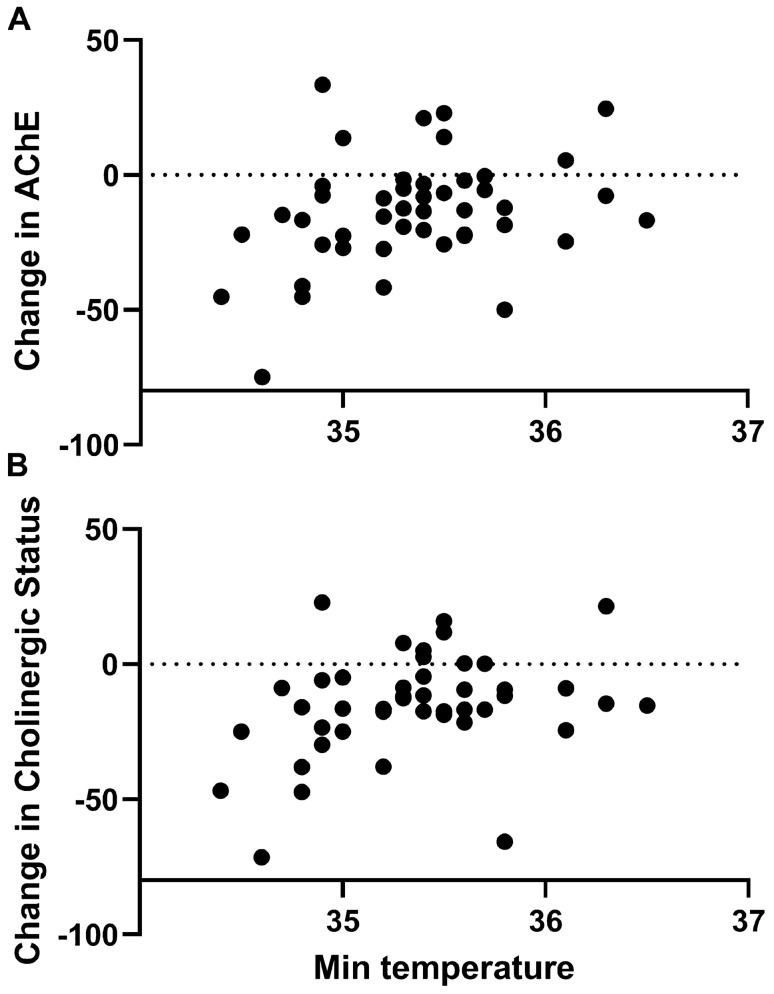
Correlation between minimal body temperature and the delta of AChE and total cholinergic status from the baseline to the end of anesthesia.

There was a positive correlation between the lowest temperature measured during anesthesia and the AChE and CS changes from the baseline values (r = 0.309 for both, p = 0.039) (Fig. 2).

This correlation remained significant even after adjustment for anesthesia length (r = 0.324, p = 0.034 and r = 0.322 p = 0.035 for AChE and CS, respectively).

## Discussion

Studies on the effect of general anesthesia and surgery on cholinesterase activity are scarce. This is the first study to show a decrease in serum cholinesterase activities during GA. Furthermore, a subgroup analysis showed a correlation between low cholinesterase activity and an increase in the need for hemodynamic support, suggesting that this biomarker can serve as a reflection of sympathetic/parasympathetic fluctuations.

A current review of the anesthesia literature suggests that heart rate variability (HRV) tracking can be used as a surrogate marker for sympathetic response and for analgesic management in clinical practice^21^. A recent study by Wujtewicz et al. used an HRV method to evaluate the relationship between the baseline autonomic tone and the vagotonic effect of a bolus dose of remifentanil^22^. Our results offer a new blood test for evaluating the cholinergic tone during anesthesia.

Our findings of a decreased cholinergic tone in general anesthesia did not clarify whether it represents a primary or secondary mechanism in the increase in resting sympathetic tone. Resolution of this issue awaits other dedicated studies. However, the cholinergic tone tends to increase with age^23^ while anesthesia decreases it^1^. Our data suggest that the changes observed with indirect measurements also occur on a molecular level. We speculate that this decrease in cholinesterase activity might be attributed to several possible mechanisms. The first is a direct effect of the medications used in anesthesia on acetylcholine, which has not been studied thus far. The second is an indirect effect of fluid shift due to a decrease in systemic vascular resistance, which causes a decrease of systemic blood pressure and venous pooling that may result in a shift of enzymes from an intravascular compartment to intercellular or intracellular compartments during anesthesia. A third option is the known increase in parasympathetic pathways during general anesthesia.

### Cholinesterase activity and hemodynamic stability

The present study demonstrated that patients with vascular disease undergoing elective surgery show significantly lower activity of AChE and CS compared to patients without vascular disease both at baseline and during anesthesia. Previous study by Arbel et al., shows that patients arriving for cardiac catheterization with lower cholinesterase activity had a higher risk for major adverse cardiac events^24^. Furthermore, Goliash et al. reported that low cholinesterase could be used as a biomarker for mortality prediction in patients with stable coronary artery disease^25^. However, the reason for the low cholinesterase activities found in these patients is not yet known. One possible mechanism could involve system exhaustion due to a high work load on the part of sympathetic pathways, or due to other illnesses, such as impaired kidney, liver or endocrine functions, even if they are not yet clinically established. Future studies will be needed in order to understand the underlying mechanism of these phenomena.

In the present study, lower cholinesterase activities during surgery and anesthesia were associated with a higher demand for drugs that support hemodynamics, such as phenylephrine. This association could be considered a reflection of the high prevalence of hypotension and the frequent need to treat hypotension to prevent postoperative morbidities^26^. We failed to show the same trend with ephedrine. One possible explanation is that most of our patients who were treated with ephedrine belonged to the non-vascular group and used lower dosage of the medication, indicating that they were more stable throughout the surgery. Another reason for a greater usage of phenylephrine among the patient in the vascular group is the connection between low cholinesterase levels and an increase in heart rate which, in the presence of hypotension, will favor the use of phenylephrine.

### Pseudo cholinesterase deficiency and its implication upon cholinergic status

BChE deficiency (also pseudo-cholinesterase deficiency) is a genetic factor that can increase the duration of a neuromuscular blockade. The most common phenotypes are “atypical” (A) and “Kalow” (K) variants (1:2800 patients), and many studies have evaluated the effect of different disease states and their correlations to cholinesterase deficiency due to this effect^27^. Evidence of a direct effect of cholinesterase deficiency on cholinergic tone, however, is lacking. Different disease states resemble cholinesterase deficiency, such as pregnancy, liver failure, kidney failure, etc^28^. It is reasonable to assume that there will be an increase in sympathetic tone in hereditary deficiency, and that these patients might therefore benefit from the testing of cholinesterase activity. We wish to emphasize that for the purpose of this study, we focused upon acetylthiocholine as the substrate of both assays (with or without BChE inhibitor—Iso-Ompa). A key advantage of the current study is our technique’s ability to focus specifically upon the ability to hydrolyze ACh, rather than BCh, highlighting AChE as more biologically relevant to the effect of anesthesia than BChE. Other studies in the anesthesia literature focused upon BChE due to its effect on muscle relaxants, such as succinylcholine and mivacurium^29^.

### Anticholinesterase and its effect on cholinergic status

Anesthesiologists use anticholinesterase drugs such as neostigmine, to reverse neuromuscular blockade. These drugs increase the accumulation of acetylcholine in both nicotinic and muscarinic receptors, enhancing the parasympathetic pathway and suppressing sympathetic pathways. In other fields of medicine, anticholinesterases are used in the treatment of myasthenia gravis, colonic pseudo obstruction, and others^30,31^. The pharmacological effect of neostigmine is short, lasting between 2.5–4 h^32^, and it can cause bradycardia up to asystole as a result of an increase in parasympathetic pathway to the heart^33^. In the current work, blood samples were taken before the administration of neostigmine, but it will be interesting to examine the cholinergic status in the postoperative period following the use of anticholinesterase medication for reversal of a neuromuscular blockade.

### Limitations of our study

Our study has a few limitations. First, the study group is relatively small, and studies on larger patient populations, especially for assessing subpopulations, are warranted. Second, baseline enzyme activity was measured at the entrance to the operating room when we might assume that the patient is anxious and so the results might not reflect the true baseline values of the patient but rather an anxious state before undergoing surgery^34^. However, this measurement was done in the same setting for all patients. Another possible limitation is genetic pseudocholinesterase deficiency and its effect on cholinergic status. To the best of our knowledge, the effect of pseudocholinesterase deficiency on cholinesterase activity to hydrolyze acetylcholine has not been studied. However, we compared the cholinesterase activity of each patient to his/her own baseline value, regardless of the genetic background. Also, further study will be needed to check the immediate postoperative period in order to evaluate the dynamics of cholinesterase's return to baseline levels. Finally, we are aware that there were a few cases with up to two risk factors for atherosclerotic disease but without any proven atherosclerotic disease in the non-vascular group, and they might potentially suffer from some level of atherosclerosis that we assumed was not severe.

In summary, the main finding of our study is that anesthesia and surgery induced a significant reduction in serum cholinesterase activity. A further subgroup analysis showed a correlation between low cholinesterase activity and an increase in the need for hemodynamic support. We speculate that patients with vascular disease are at increased risk for hemodynamic support because their basal cholinesterase activities are lower than those of non-vascular patients. Further research is needed on larger numbers of patients with the aim of understanding the effect of severe atherosclerosis on decreased cholinesterase activity during anesthesia, and the implication it might have on perioperative care as a possible biomarker before and during surgery.
